# The use of the Roy Adaptation Model in clinical nursing care of an adolescent diagnosed with type 1 diabetes (case report)

**DOI:** 10.3389/fcdhc.2026.1721420

**Published:** 2026-04-16

**Authors:** Ecem Oksal Güneş, Senay Cetinkaya

**Affiliations:** 1 Cyprus Aydın University, Girne, Cyprus; 2 Cukurova University, Adana, Türkiye

**Keywords:** adolescent, diabetes, nursing care, Roy adaptation model, type 1 diabetes

## Abstract

Adolescence is considered one of the most difficult periods in life due to the physical, cognitive, emotional and social changes that occur. With the developmental characteristics of adolescence and the existence of a chronic disease such as type 1 diabetes (T1D), adolescents may face problems such as a decrease in self-esteem, feeling different from their peers, and not being able to be included in peer groups. In this case report, nursing approaches to improve the physical, psychological, and psychosocial adaptation of an adolescent diagnosed with T1D were implemented based on the Roy Adaptation nursing model. In line with data obtained (with permission) from a 14 -year-old patient with T1D, pain, nutritional insufficiency, hyperglycemia, fatigue, decreased social interaction, lack of knowledge, anxiety, inadequacy in individual coping, ineffective role performance and impairment in social interaction were diagnosed and appropriate nursing interventions were implemented. In this context, the patient was given counseling and nursing care on the complications of diabetes, insulin administration training, and diet training, ensuring the organization of her self-concept, roles, and responsibilities, eliminating the lack of information regarding the diagnosis of diabetes, and ensuring compliance with the diagnosis of diabetes. At the end of the nursing interventions, the patient’s pain was relieved. Since nausea is the cause of the patient’s “nutrition less than the body requirement” problem, interventions to relieve nausea have yielded positive results. Fatigue decreased and the patient was more willing to perform activities. The patient’s lack of information about her ilnesses was addressed. Despite all nursing interventions about the patient’s role function and mutual interaction areas, she still expresses the changes caused by her illness in her school life with negative evaluations. In this case report, it was seen that the use of the Roy Adaptation Model was appropriate and beneficial, considering that the most common problems among adolescents hospitalized for T1D are physical, psychological, and psychosocial adaptation to the illness.

## 1 Introduction

A chronic metabolic condition known as type 1 diabetes (T1D) is defined by a lack of insulin hormone in the blood, which is caused by the autoimmune destruction of pancreatic beta cells (1). The Ministry of Health report states that the prevalence of T1D in children under the age of 18 in Turkey is 0.75/1,000, whereas the incidence is 10.8/100,000. The preschool (4–6 years) and adolescent (10–14 years) periods are the two periods when T1D peaks (2). T1D constitutes a significant and growing burden in pediatric health service provision, with an increasing incidence rate of 3.4% annually in most European countries (3). A particularly difficult transition period for children living with T1D and their families is the adolescence period, a time where deterioration in metabolic control has been observed (4). Multiple factors (e.g., pubertal insulin resistance, risk-taking behaviors, and poor treatment adherence) contribute to the deterioration in metabolic control as adolescents mature (5). The management of diabetes in the teenage group presents a more complex set of challenges given the variety of physiological, social, and emotional changes that occur between childhood and adulthood, including puberty, peer pressure, the desire to be “normal”, identity formation, and social relationships (6). Given the importance of quality of life in diabetes, various nursing models could be used to enhance this parameter among patients with diabetes. One of the efficient nursing models in this regard is the Roy Adaptation Model (RAM). The RAM, with the aim of performing nursing care measures to improve adaptation responses in four psychological, self-concept, role function, and independence modes, is among nursing care patterns that extensively deal with the issue of adaptation in patients with chronic diseases (7).

In nursing practice, the use of models is important in determining the basic concepts and the relationship between concepts, defining the problems in practice, and developing solution proposals (8). The RAM is a widely used model in determining the conceptual framework of nursing. The RAM can be used in all areas of nursing. Roy sees the individual as an adaptive system in constant interaction with his internal and external environment. According to the RAM, the aim of a nurse should be to increase the individual’s harmony and wellbeing (9). In this context, school nurses in particular could act as a true manager of care in heterogeneous contexts, in terms of both resources and management (10, 11). With advances in technology in diabetes care, >90% of school nurses have experienced caring for children with insulin pumps and continuous glucose monitoring (CGM) (March et al., 2023) (12). School nurses should be the key coordinator and care provider, professionally responsible for acquiring and maintaining knowledge and competencies in diabetes management (13). Effective collaboration among educators, administrators, and support staff ensures a positive school culture that nurtures academic success and personal growth (14). Ensuring their full participation in academic and social activities requires an inclusive approach that prioritizes both their medical needs and emotional wellbeing. In this context, the role of a school nurse is essential, as they are responsible for monitoring and supporting the diabetic student, working to create a friendly and safe environment for them in the school setting (15, 16).

The RAM is based on the concepts of “System” and “Adaptation” (17). Holistic nursing care is important in the treatment of diabetes, which affects many systems. In nursing, which is a professional discipline, the scientific knowledge source of care practices is constituted by concepts and theories, and these practices are carried out with nursing models. The use of the model systematizes care, allowing nurses to focus on nursing practices rather than medical practices. At this point, care was provided for the adolescent diagnosed with T1D using the RAM, which is one of the nursing models widely used in nursing and focuses on the individual’s adaptive system.

### 1.1 The use of the Roy Adaptation Model in solving the adaptation problems of adolescents with T1D

This case report was conducted to evaluate the qualitative data of an adolescent with diagnosed T1D according to the RAM. Data were collected after necessary explanations were made to the adolescent and her legal guardian, and their written consent was obtained. The principle of confidentiality was observed and ethical principles were adhered to in the case report. Based on the RAM, the biological, psychological, and social integrity of the adolescent who received nursing care for 7 days was evaluated.

## 2 Case description

C.G. is 14 years old, was diagnosed with T1D 7 years ago, and came to follow-up every 3 months. She was last admitted to the hospital with severe abdominal pain and vomiting. Her liver enzymes were said to be high, and she was hospitalized for 1 week and discharged. After discharge, her blood sugar was high, she was hospitalized again for 1 week, and her blood sugar was regulated. The last reason for her admission to the hospital was severe abdominal pain, constipation, vomiting, and weakness. For this reason, she was referred to the Pediatric Endocrine Department. The Pediatric Endocrine outpatient clinic referred the patient to the emergency room. The first blood glucose measured in the emergency room was 600 mg/dL, insulin and fluid were given, blood sugar was measured at 111 mg/dL, and she was admitted to the Pediatric Endocrine service after being stabilized.

### 2.1 Child health and diseases nursing care plan form

#### 2.1.1 Introductory ınformation

Child’s age: 14.

Gender: Female.

Education status: Secondary school 8th grade.

#### 2.1.2 Perception and management of health

Health information (reason for hospitalization): The patient who has had T1D for 7 years and comes for check-ups every 3 months presented to the hospital with complaints of abdominal pain and vomiting.

#### 2.1.3 Prenatal

Second living child from pregnancy.

#### 2.1.4 Postnatal

Diseases: Urinary tract ınfection, thalassemia carrier.

Immunization: Fully vaccinated.

Mode of delivery: Normal birth.

Birth weight: 2,295 g.

Medication used regularly: Insulin.

Growth and development: The percentile of weight according to age was measured between 10% and 25%, and the percentile of height according to age was measured between 3% and 10% percentile.

Nutrition–metabolic function: Nutritional status: oral, natural, self-fed, salt-free diet, has little appetite, and has experienced a decrease in weight. She has nausea but no vomiting at the moment. She does not eat much during the day during her 24-h diet.

Urinary excretion

Urine: pH 5.5, clear, light yellow.

Bowel emptying: Bowel sounds are normal, the abdomen is soft, and toilet habits are regular.

Musculoskeletal–neurological system

No joint limitations, no fractures, no dislocations, no decrease in muscle coordination and strength, and no anomalies.

Cardiovascular system

Activity tolerance: She stated feeling weak and exhausted. Her pulse was normal and she had no tachycardia.

Respiratory system

Her breathing was regular; she did not have dyspnea or cyanosis. Abnormal breathing sounds were not observed.

Sleep–rest

She said she did not sleep at night.

Daytime sleep: She had a short sleep of 20–30 min.

Mental–perceptual functıon

Eyes: Normal, had a corneal reflex.

Ears: Normal.

Skin/touch/sense of feeling: There was no change in the perception of heat/cold.

Mouth: Mucous membranes were normal.

Skin turgor: Normal.

Pain sensation: Abdominal pain is present.

Memory

She thought she would fail at school because she was in the hospital.

Self-perceptıon and self-concept

She was feeling exhausted and unsuccessful. She stated that she was unable to fulfill the responsibilities given to her at school.

Roles–relationships

The family has accepted the disease, and there is parental support. She was an active child before the illness; after the illness, she looked tired and exhausted all the time.

Coping with stress

Her facial expression is tired and sad; she is stressed because she is in the hospital, and her parents are supportive.

Values/Beliefs

The family did not do anything special in terms of tradition.

Sexuality and reproduction

She has a menstrual cycle and she has a regular perimenstrual period of abdominal pain.

Vital Signs and Laboratory Findings

Body temperature: 36.5°C, pulse: 100/min, respiration rate: 26/min, blood pressure: 110/70 mmHg, height: 117 cm, and weight: 37 kg. Hourly follow up of diabetic ketoacidosis (**Table 1**) and monitoring of laboratory tests (**Table 2**) were performed.

**Table 1:** DKA (diabetic ketoacidosis) follow-up chart.

Hour	Glucose	pH	CO_2_	HCO_3_	Be	K	N	K	NA	Dext	Insülin	Acetone
21.00	120	7.40	35	22.8	−1.9	4.3	139	20	100	2.3	0.1	+++
22.00	110							20	100	2.3	0.1	
23.00	106							20	100	3.3	0.1	
24.00	64	7.41	34.6	22.7	−2			20	100	6.6	Stop	
01.00	111					3.8	138	30	100	10	0.075	+

**Table 2:** Laboratory findings.

Values	Reference range
Glucose: 386	70–100
Total cholesterol: 291	291
BUN: 14	8–20
Na: 135	135–145
K: 5.4	3.6–5.2
Total Ca: 9.7	8.9–10.3
Inorganic P: 4.4	
Mg: 1.6	1.8–2.5
Total protein: 6.8	
Total albumin: 3.7	2.7–4.8
Hdl: 42.3	40–60
Triglycerides: 367	<200
Ldl: 175	<160
HbA1c: 10.90	4.0–6.0
Urinalysis pH 5.5, clear, light yellow	

## 3 Nursing process according to Roy

### 3.1 Assesment of behaviour

- Observation and interview were used to diagnose her behavior. She was first observed sitting on her bed with her feet drawn to her stomach and stated that she had abdominal pain.
- In addition, she said that she did not want to eat in the hospital and refused to eat because the food made her nauseous.
- She looked tired and exhausted, rejecting her mother’s offer to walk around the ward, expressing that she did not want to get out of bed and that she was sluggish. She just wanted to play games with her tablet computer in bed.
- When I ask how are your classes going, she said; “It was good but, because I’m here, I can’t do my homework, I can’t attend classes, I can’t study my exam”. She was a successful student, but she seemed anxious and sad that she could not attend classes, and she expressed her sadness in words.
- She knew the indications and side effects of the drugs she was using, but she was not serious about what could happen if she did not use them. Her mother stated that when she wanted to bring the patient for check-ups, she often refused to come, her blood sugar had improved, and she no longer wanted to go to the hospital.
- She stated that she wanted to get out of the hospital as soon as possible and go back to school and that she missed her friends. She also complained that her friends did not come to see her, and she was upset about it.

### 3.2 Assesment of stimuli

The reason for her hospitalization is T1D, an increase in blood sugar, and ketoacidosis. Therefore, the most important focal stimulus that is effective in the emergence of existing or potential problems of C.G. is T1D; not getting enough sleep and rest, experiencing pain, fear of lifestyle changes, and being in the hospital are among the other focal stimuli.

Among the contextual stimuli are “not taking her medications regularly, side effects of ketoacidosis, being in a hospital environment, interventions, and being away from home and school environment”.

The residual stimuli that cause C.G.’s problems include hospital meals, not coming to regular check-ups, treatments, and not being able to fulfill the responsibility of lessons and homework.

### 3.3 Determining nursing diagnoses

Nursing diagnoses were made after observing behaviors, identifying problems, and determining the stimuli that could cause these problems.

Nursing diagnoses are as follows:

- Pain
- Imbalance in nutrition, nutrition less than the body needs
- Fatigue, weakness
- Anxiety
- Lack of information
- Ineffective role performance
- Impairment in social interaction

### 3.4 Setting goals

Our first goal is for the patient to express that her pain is gone, then to express that her nausea is gone and to be able to eat her favorite foods, to reduce her fatigue and to participate in activities, to reduce her anxiety and tension, to eliminate her lack of knowledge, to express the changes caused by her illness in school life with positive evaluations, and to increase her social interaction.

### 3.5 Intervention

The patient’s identified problems are solved with appropriate nursing interventions through appropriate nursing diagnoses. Appropriate nursing interventions applied to the patient are given in **Table 3**.

**Table 3:** Nursing care plans based on the Roy Adaptation Model.

Physiologic area	
Behavior	Pulling her feet to stomach and expressing that she has pain.
Focal stimulus	Hyperglycemia, ketoacidosis
Contextual stimulus	Not taking her medication regularly
Residual stimulus	Failure to come for regular check-ups
Nursing diagnosis	Pain
Aim	Verbally/non-verbally expressing that the pain is gone, targeting a lower pain score
Nursing intervention	The location, intensity, onset time, and increasing and decreasing factors of pain are evaluated together with the patient. Her emotional reactions and coping style to pain are assessed and monitored. The increase and decrease of pain in daily living activities are determined by discussing with the patient and unnecessary movements are avoided. Anti-inflammatory, analgesic drugs are prescribed, and the expected effect is explained. While the drugs are administered, arrangements are made in a way that does not interrupt sleep and rest. When she gets up in the morning, she is allowed to take a warm shower or bath. 7. Directed to leisure activities, daily rest periods are planned.
Assessment	She verbally stated that her pain was gone.

Physiologic area	
Behavior	She stated that she could not taste the food, and that she did not want to eat because it increased her nausea.
Focal stimulus	Ketoacidosis, Hyperglycemia
Contextual stimulus	Side effects of ketoacidosis
Residual stimulus	Anxiety about being hospitalized, the appearance of food
Nursing diagnosis	Imbalance in nutrition, nutrition less than the body needs
Aim	Controlling nausea, preventing weight loss by offering favorite food alternatives
Nursing intervention	Regular weight measurements are taken and recorded. It is ensured that the foods she likes are coordinated with the dietitian and written into the regimen and come from the cafeteria. Food is given warm, not hot or cold. Nausea is controlled before meals, and tiring care practices are avoided. The patient is helped to rest before meals. Food was given in small, frequent portions, and drinks were administered via straw. Interventions to reduce nausea are taught (ventilation of the environment before feeding, loosening clothes, giving a sitting or semi-sitting position, restricting liquid drinking with food, and avoiding fatty or pulpy foods).
Assessment	It has been observed that the patient’s nausea is controlled and she starts to eat her favorite foods

Physiologic area	
Behavior	She states that she does not want to get out of bed; she does not want to get up even for her daily needs.
Focal stimulus	Insufficient sleep and rest
Contextual stimulus	Being in a hospital environment, nursing and medical interventions
Residual stimulus	Treatments and interventions
Nursing diagnosis	Fatigue, weakness
Aim	To reduce fatigue and to ensure the participation of the person in activities.
Nursing intervention	Asked to record the activity status and hourly fatigue status during the day. By evaluating the findings together, the situations in which she feels the most tired and dynamic are revealed. Situations in which she feels energetic are supported. During these periods, it is ensured that she does activities for her own care. Appropriate activity/recreation programs are developed. Visitor planning is made and everyone is interviewed one by one. Information about effective coping methods (relaxation, talking-commiseration, etc.) is given. Sleep patterns are improved. The systematic effect of rest on reducing emotional stress and joint problems is explained.
Assessment	She stated that her fatigue decreased and she was more willing to do activities.

Self-concept area	
Behavior	Feeling of nervousness and anxiety (express that she will fail the class)
Focal stimulus	Diabetes (chronic disease)
Contextual stimulus	Pain
Residual stimulus	Change in role performance in school life
Nursing diagnosis	Anxiety
Aim	Providing relief by reducing the feeling of nervousness and anxiety
Nursing intervention	She is encouraged to share her feelings and fears. By allocating time, her self-perception is evaluated and she is supported to express her thoughts. It is allowed to express her thoughts on the performance of the role. Past coping methods are evaluated, and the appropriate method is used. In cooperation with the teacher, she is helped to follow her lessons. Helps to communicate with friends and keep track of homework.
Assessment	The teacher was contacted, the responsibilities of the homework were learned, and C.G. was encouraged to do her homework. C.G. stated that she was relieved after doing her homework.

Self-concept area	
Behavior	Not coming to regular check-ups, not using her medications regularly
Focal stimulus	Fear of changes in lifestyle
Contextual stimulus	Inability to integrate the treatment plan into daily activities
Residual stimulus	Uncertainty about her future life
Nursing diagnosis	Lack of information
Aim	Eliminating the lack of knowledge
Nursing intervention	The patient’s knowledge about her illness and treatment is assessed. The patient and her family are informed about their health problems in a way they can understand. The patient is informed about the effects and side effects of the drugs in the treatment plan. It is emphasized that the drugs should be used as recommended and that the healthcare team should be notified when side effects develop. Support is provided for goal creation and problem solving. The importance of regular use of the drugs in the treatment plan is emphasized and information is given about the complications that may occur if they are not used.
Assessment	The patient’s knowledge about the disease and drug use was evaluated and the lack of information was eliminated.

Role function area	
Behavior	She states that she wants to go to school but cannot follow her lessons
Focal stimulus	Ketoacidosis, hyperglycemia, pain
Contextual stimulus	Inability to integrate the treatment plan into daily activities
Residual stimulus	Failure to fulfill the responsibility of courses and homework
Nursing diagnosis	Ineffective role performance
Aim	Patient’s positive evaluation of the changes in her school life caused by her illness
Nursing intervention	The changes experienced by the patient and the family, occupational and social roles, along with the disease process are evaluated. She is allowed to explain her thoughts about the performance of the role. She is directed to make arrangements in line with the role changes she is experiencing. She is supported to continue with the usual roles and activities she can do. She is helped to follow her lessons in cooperation with a teacher.
Assessment	She expresses the changes caused by her illness in her school life with negative evaluations.

Mutual interaction area	
Behavior	Stating that she could not see her schoolmates and that she missed them
Focal stimulus	Being in hospital
Contextual stimulus	Hyperglycemia, ketoacidosis
Residual stimulus	Not being able to see friends
Nursing diagnosis	Impairment in social interaction
Aim	Expressing increased social interaction
Nursing intervention	She is encouraged to share her feelings. She is asked to identify situations that cause deterioration of social communication. Ways to initiate social interaction with the patient is discussed. She is allowed to make phone calls with her friends. Encourage the experimentation of new social behaviors.
Assessment	She was made to make a phone call to her friend. She felt better, but she expressed sadness that her friends did not come to see her.

Assessment.

- She verbally stated that the pain was gone.
- On the third day of the interventions, she wanted to participate more in the activities.
- After speaking with her teacher and learning about homework, she stated that she felt better after completing her homework.
- Her lack of knowledge was resolved, and she was informed about what could happen if she did not take the drugs.
- She was still sad because she could not see her friends.

## 4 Biopsychosocial adaptation areas of the case based on the Roy Adaptation Model

### 4.1 Physiological area

1. She was in pain and she was sucking her stomach in. **(Pain)** Weight gain was reduced, her percentile values were low, she refused to eat food from the cafeteria, and she expressed nausea.
2. (Nutrition less than the body requirement)
3. She looked tired and sluggish, refusing to walk along the hallway, saying she just wanted to stay in bed and lie down. (Fatigue, weakness) (**Table 3**).

### 4.2 Self-concept area

1. She complained and was worried about not being able to do her homework and not being able to attend school. (Anxiety)
2. She knew about the side effects and indications of the drugs, but she did not know the complications that could happen if she did not take her medications. She did not know about the importance of regular check-ups. (Lack of information) (**Table 3**).

### 4.3 Role function area

1. She stated that she wanted to go to school, and if she was absent from classes, she would fail the class.
2. She stated that it was exam time and she would not be able to take the exam and the result would be bad. (Ineffective role performance) (**Table 3**).

### 4.4 Mutual ınteraction area

1. She stated that she could not see her friends. She missed her friends. (Impairment in social interaction) (**Table 3**).

## 5 Discussion

### 5.1 Roy’s perspectives on clinical and allied nursing ınterventions based on Roy adaptation theory

#### 5.1.1 Physical adaptation

This study explored the effects of nursing interventions grounded in the RAM on adaptation outcomes in children living with diabetes. Overall, the findings indicate that RAM-based interventions can positively influence physiological regulation, psychosocial adjustment, and adaptive behaviors in pediatric diabetes care. A core aim of the RAM is to enhance the individual’s ability to respond adaptively to physiological stimuli. Structured nursing interventions, such as individualized education on insulin administration, blood glucose self-monitoring, diet planning, and tailored physical activity guidance improve glycemic control and reduce acute glycemic fluctuations in children with T1D. For example, Kara and Aydın (2021) found that a RAM-based tailored education program significantly improved HbA1c levels and reduced the frequency of hypoglycemic events compared to standard care. These findings align with Roy’s conceptualization that targeted support within the physiological mode strengthens biological integrity and promotes homeostasis (18). In a prospective study involving participants aged 8–18, repeated self-management education focused on glucose monitoring, insulin adjustment, and lifestyle awareness and demonstrated improved glycemic variability outcomes. Post-education assessments showed more frequent glucose monitoring, improved time in the target glucose range (70–180 mg/dL), and better hypoglycemia management skills compared to pre-education status. These improvements reflect decreases in acute glycemic fluctuations, especially hypoglycemia episodes, which are critical for pediatric safety and long-term control (19). In parallel research, pediatric patients initiating continuous subcutaneous insulin infusion (CSII) who received tailored diabetes education showed not only increased adherence to treatment but also significantly improved glycemic indices (e.g., greater time in range and reduced HbA1c levels) over 6 months. This suggests that when education is adapted to technological and individual readiness, children and adolescents are better able to normalize glucose levels and reduce hyperglycemia and glycemic variability (20). A systematic review study concluded that both students and educators benefit from more efficient management in settings where school nurses are present; however, it also noted areas that need to be re-evaluated, particularly regarding the management of T1D using technological devices; healthcare organization is often lacking, compounded by deficiencies in school systems where professionals frequently lack specific training, especially for emergency situations. In nearly all cases examined in the study, parents remain the primary caregivers for students, revealing critical gaps in modern, integrated care systems. As a result of this systematic review, they stated that “Effective student integration necessitates addressing both the health and social aspects, urging health managers to develop specific care pathways for students of all ages and socioeconomic backgrounds living with T1D and using technological support.” (21).

#### 5.1.2 Self-concept adaptation

The self-concept mode in RAM encompasses psychological and spiritual dimensions of adaptation. Children with diabetes often face emotional stressors such as fear of injections, feelings of difference from peers, and worries about long-term complications. Nursing interventions that include psychological support, motivational interviewing, and age-appropriate disease education can improve children’s confidence in self-management and reduce diabetes-related distress. For instance, Yılmaz and Koç (2019) reported improved self-efficacy and more positive illness perceptions among children who received RAM-based supportive nursing care, compared to control groups. These improvements reflect enhanced internal adaptive mechanisms and support Roy’s assertion that nurturing self-concept contributes to overall adaptation (22).

#### 5.1.3 Role function and mutual adaptation

The role function mode focuses on fulfilling expected social roles. Chronic illness in childhood can disrupt school participation, peer relationships, and leisure activities. Studies investigating RAM-guided nursing interventions emphasize collaborative care planning that includes school staff, caregivers, and children themselves. Demir and Arslan (2022) observed that children who participated in such interventions maintained higher levels of school attendance and engaged more fully in age-appropriate activities. Through reinforcement of adaptive behaviors and environmental support, these interventions facilitate successful integration of the diabetes management role with other developmental roles, consistent with RAM’s focus on adaptive role function (23). The interdependence mode within RAM highlights the importance of supportive relationships. Family involvement is a key component of diabetes care, particularly in children. Nursing interventions, which include family education, caregiver coaching, and communication strategies, improve adherence to treatment plans, collaborative problem solving, and emotional support. For example, Surucu et al. (2021) demonstrated that family-inclusive RAM-based interventions improved parent–child team work in diabetes management, leading to better adherence and fewer clinical complications. Enhanced interdependence fosters adaptive social support networks, reinforcing the child’s capacity to meet daily diabetes management demands (24).

## 6 Conclusion

In the case report examined according to the RAM, nursing interventions were applied in four stages according to the adaptation areas. *Nursing diagnoses related to the physiological field*: Nursing diagnoses of pain, less nutrition than the body’s needs, fatigue, and weakness were discussed and interventions were planned. By evaluating the patient’s emotional reactions to pain and the way of coping, interventions were planned and the patient’s pain was relieved after the application. Since nausea is the cause of the patient’s “nutrition less than the body requirement” problem, first of all, attempts to relieve nausea and diet planning attempts by coordinating the patient’s favorite foods with the dietitian have yielded positive results. It was observed that the patient’s nausea was controlled and she started to eat her favorite foods. The patient was tired and sluggish due to lack of regular sleep and inability to rest. By planning visitor and treatment hours and developing appropriate activity/rest programs, the patient was allowed to rest and sleep. She stated that her fatigue decreased at the end of these interventions and she was more willing to do the activities. Information on metabolic control (recognizing the symptoms of hypoglycemia and hyperglycemia and learning how to intervene), insulin administration (the right dose in the right time period), insulin storage conditions, diabetic foot, and nutrition (three main meals and three snacks) was provided, and positive results were obtained from the patient. *Nursing diagnoses related to the self-concept area*: Diagnoses of anxiety and lack of knowledge were discussed. The patient’s concerns about the disease were relieved and she stated that she was relieved. *Nursing diagnoses related to the role function concept area*: Diagnoses of inadequacy in individual coping and deterioration in the continuation of the family process are discussed and the patient agreed to continue with the usual activities she can do, but she expresses the changes caused by her illness in her school life with negative evaluations. *Nursing diagnoses related to the mutual ınteraction area*: Diagnosis of impairment in social interaction was discussed. She is asked to identify situations that cause deterioration of social communication and she was encouraged by the experimentation of new social behaviors. She was allowed to make a phone call to her friends. She felt better, but she expressed sadness that her friends did not come to see her.

These findings reinforce the importance of structured, individualized nursing care as a fundamental component of pediatric diabetes care; this conclusion aligns with updated clinical practice guidelines that emphasize personalized care rather than one-size-fits-all approaches. The collective evidence suggests that nursing interventions framed by the RAM can strengthen adaptive responses across multiple domains in children with diabetes. However, several limitations exist in the current literature. Many studies utilize small sample sizes, short follow-up periods, and variations in intervention content and delivery, which challenge the generalization ability of findings. Future research should prioritize randomized controlled trials with larger samples and standardized intervention protocols to determine the long-term effectiveness of RAM-based nursing care in pediatric diabetes populations.

In conclusion, this body of work supports the utility of the RAM as a theoretical framework for nursing interventions aimed at enhancing both physiological and psychosocial adaptation in children with diabetes. By addressing multiple adaptive modes, RAM-guided care contributes to improved health outcomes and quality of life, underscoring its relevance to contemporary pediatric nursing practice.

## Data Availability

The original contributions presented in the study are included in the article/supplementary material. Further inquiries can be directed to the corresponding author.
